# Effects of a 3-factor field intervention on numerical and geometric knowledge in preschool children

**DOI:** 10.1371/journal.pone.0290956

**Published:** 2023-11-16

**Authors:** Hernando Taborda-Osorio, Yenny Otálora

**Affiliations:** 1 Department of Psychology, Pontificia Universidad Javeriana, Bogotá, Colombia; 2 Faculty of Psychology, Center for Research on Psychology, Cognition and Culture, Universidad del Valle, Cali, Colombia; University of New England, AUSTRALIA

## Abstract

The main aim of this study was to develop and test the effects of a field math intervention program on both number and geometry knowledge. The intervention was developed based on three basic skills previously associated with mathematical performance: symbolic number knowledge, mapping processes and spatial reasoning. The participants were 117 preschoolers from six schools in Cali and Bogotá. The children were assigned to an intervention group (N = 55) or a control group (N = 62). The intervention lasted 11 weeks with 3 sessions per week where the children participated in different game-based activities. Tests of numerical and geometric knowledge were administered before and after the intervention. The effects of the intervention were tested twice, immediately after the program ended and six months later. The results show that the children in the intervention group improved more than the control group in both number and geometry. The second posttest revealed a significant intervention effect for geometry, but not for numerical knowledge. The implications of these mixed patterns of results are discussed in the paper.

## Introduction

Research on mathematics education has shown the relevance of developing interventions in early childhood [1,2]. Preschool-aged children are sensitive to new intervention programs in diverse settings and through different materials [2–4], showing better mathematical performance over time compared to children in both business-as-usual programs and active programs. The importance of early mathematical interventions is supported by evidence showing an association between early math performance and later mathematical outcomes [5,6]. For instance, preschoolers who are low achievers in mathematics still struggle one and two years later in formal tests [6]. Additionally, the period between 5 and 7 years of age seems to be critical in the development of the emergence of both geometric and numerical formal knowledge [7,8]. Therefore, impacting this age range through appropriate intervention programs may have an effect on foundational knowledge and abilities for the school years.

Current studies on the development of mathematical thinking have provided researchers with a great deal of insight into those factors that can promote critical changes in the learning of both formal and informal mathematical knowledge. The literature review shows evidence of at least three of these factors: 1) the learning of symbolic number knowledge [1,9,10]; 2) the development of spatial reasoning [8,11,12]; and 3) the learning of the mapping between symbolic and non-symbolic number systems [3,4,13,14]. Children experience changes in all three factors from early in the preschool years and these changes appear to be associated with mathematical performance in concurrent and predictive ways. Importantly, the effect on mathematical performance extends to arithmetic skills as well. We breakdown each of these factors below.

S*ymbolic number knowledge* refers to the knowledge of Arabic numerals and spoken number words [10]. Some authors refer to this knowledge as the symbolic number sense to point out its foundational nature in the acquisition of more complex mathematical skills [6]. For example, at 6 years of age children’s number identification skills predict arithmetic performance a year later [9]. This effect was found above and beyond the children’s non-symbolic numerical knowledge. Both procedural and conceptual counting skills are also good predictors of arithmetic skills [15,16]. However, number skills such as number identification and number comparison have been shown to be better predictors of mathematical achievement in first grade than counting skills [17]. Additionally, critical to this factor is children’s ability to connect quantities with Arabic symbols [1,18,19], for instance, representing a set of four points with the number word “4” or the spoken number word “four”. Several studies have shown the causal relationship between symbolic number knowledge and arithmetic skills [6,20].

*Spatial reasoning* refers to the ability to reason about objects and their spatial properties [8]. It has been proposed that spatial abilities can be malleable [21] and are fundamental for later interest and success in STEM disciplines [21–23]. In particular, the last 10 years of research have shown a growing number of studies on the relationship between spatial and mathematical thinking [24–26]. This association has been observed in both preschool and school-age children. For example, better mental rotation skills are related to better arithmetic skills in 6- to 8-year-olds [27]. Spatial assembly performance with three-dimensional blocks is also associated with informal mathematical skills in 3-year-olds [28], and preschoolers’ performance in spatial scaling tasks is associated with performance in proportional reasoning tasks [29]. In a recent study [8], only some spatial transformation skills such as mental rotation and spatial scaling predicted mathematical achievement two years later in second grade. However, in this same study, spatial tasks with an allocentric frame of reference (e.g., mental transformation and perspective taking) were associated with children’s performance in geometry. The causal association between spatial reasoning and mathematical performance is more controversial; some intervention studies show either a weak or no effect [2,30–33], while others show a robust effect [19,27,34,35].

*Mapping* refers to the developmental process of alignment between symbolic and the non-symbolic number systems, which follows gradual progress from the preschool years and beyond [36]. A wide variety of studies have provided evidence for a small but statistically significant effect of the non-symbolic number system acuity on math achievement [37–39]. However, more recent evidence suggests that the mapping between symbolic and non-symbolic math knowledge is a better predictor of mathematical achievement, and especially in formal mathematics, than the acuity of the non-symbolic number system [10,14,40–42]. Two types of mapping tasks have been used in these studies: the number estimation task and the number line estimation task [43]. Several recent studies have shown positive effects of interventions through number board games, in which, among other mathematical skills, children train the acuity of the mental number line [3,4,44,45]. Another study directly trained preschoolers in a number line estimation task, that showed positive effects on arithmetic performance [46].

Although important for educational purposes, most of the aforementioned evidence has been collected in controlled laboratory settings. It is unclear to what extent these findings can be translated into successful instructional practices in real school settings. This is particularly true for spatial reasoning and mapping processes, whose effects on mathematical achievement are as yet less understood. In one notable exception [13], a very large field study conducted in India with preschool children, found a short-term effect on mathematics performance through an intervention based on intuitive (non-symbolic) mathematics and intuitive spatial reasoning, but found no reliable effect one year later. All the activities developed in this study were challenging and fun games for children based on the use of inexpensive, easy-to-make manipulatives and prints. However, the format of some of the tasks still bore a strong resemblance to typical lab activities. Despite this, the latest study highlights the importance of promoting the design of play-based activities that young children may find more fun, as well as the importance of implementing interventions in school settings to reinforce previous lab findings.

Another issue that makes it difficult to assess the effect of the above interventions is that mathematical knowledge is measured in many ways. Some studies take as reference both geometry and numerical knowledge [13], others only arithmetic [35], while others measure both formal and informal math [14]. However, as highlighted above, the preschool years are critical in the emergence of formal mathematics. Therefore, it is vital to better understand the effect of an early intervention program on the different dimensions that make up mathematical knowledge, including formal math. Furthermore, the distinction between formal and informal math seems to be particularly relevant for children with mathematical difficulties (MD). Some studies report that children with MD show lower performance in formal math than in informal math, both in school [47,48] and preschool-aged children [49]. This difference in performance increases over time, and therefore children with MD continue to have difficulty with formal math activities in the early years of school education [49].

Furthermore, several studies have also shown that low-income preschool-aged children can benefit from early math interventions [1,3,4]. These studies have highlighted the type of materials and tools used to help these children progress through the interventions. For instance, a study [1] implemented activities and materials focused on building connections among varied representations of number and quantities, including iconic, enactive, and symbolic. Similarly, other studies [3,4] showed the relevance of utilizing games that resemble the mathematical structure of mathematical concepts and processes such as number line estimation. They found that children who played linear board games that align with the number line representation demonstrated better mathematical performance than children who played color board games [3] or circular board games and other numerical activities [4].

### Present study

The main goal of the present study was to develop and test the effects of a field math intervention program on both number and geometry knowledge. The intervention aimed to improve the math skills of Colombian preschool-aged children with respect to typical educational practices by using learning activities based on the three factors mentioned above in preschool classrooms: symbolic number knowledge, spatial reasoning, and mapping processes. A considerable body of evidence indicates that all three factors contribute to improving children’s numerical skills [9,14,27,35]. However, most of this evidence comes from correlational research and interventions in controlled laboratory settings. Therefore, evidence is needed from school settings on the importance of these three early competences in promoting math learning. To our knowledge, this is the first reported intervention that seeks to integrate these different factors in a school setting using game-based and contextualized activities. Particularly important in this intervention is the effect of spatial reasoning on geometry knowledge. Some studies have found an association between both skills in children [8,50]. One possibility is that solving some geometry items requires the use of mental transformation skills, such as spatial visualization and mental rotation. Therefore, our hypothesis is that training children in spatial reasoning may have an effect not only on numerical knowledge, but also on solving simple geometry tasks.

To promote better contextualized activities for the school setting, the intervention program involved engaging and challenging tasks embedded in learning environments that were created and delivered under carefully defined pedagogical criteria grounded on prior literature in the field [3,8,13,18,51,52]. These tasks were based on the use of a variety of tools to represent concepts including verbal prompts, stories, visual representations, manipulatives, TIC, and the body; also, the activities involved both explicit and implicit feedback and scaffolding (see the methods section for more details). Thus, we aimed to adapt typical lab activities to a school context through the creation of new game-based situations, implementing pedagogical practices that somewhat resemble the more typical activities of preschool teachers and thus increasing the chances of scaling up a successful intervention program. Below, in the “intervention program” section, we present the details of these activities.

In addition, we measured both formal and informal children’s math performance to gain insight into the differential effects of the intervention. As mentioned above, few intervention studies explore systematically the effectiveness of new math learning programs through this distinction. In current research, informal math knowledge refers to concepts and skills learned before entering school and acquired in the children’s everyday experience, such as counting and solving simple word problems. Formal math knowledge refers to the skills to understand and use conventional math symbols, typically learned in school settings, such as solving arithmetic problems and numerical transcoding. All three factors that make up the intervention program have shown effects on formal math skills in preschool-aged children. Therefore, our hypothesis is that the intervention should have an effect on both informal and formal math knowledge.

The distinction between formal and informal is less clear in early geometric knowledge. However, a parallel distinction has been made between intuitive geometry and academic geometry [53]. Intuitive geometry refers to a type of implicit mathematical knowledge independent of education and learnt through informal experiences; such as, concepts of symmetry, congruence, points, lines, parallelism, and metric properties, among other concepts [54,55]. Academic geometry refers to a type of explicit mathematical knowledge, that is more reliant on school teaching and the learning of cultural symbolic systems. Thus, academic geometry requires the learning of definitions and the ability to solve geometrical problems. An example of this knowledge in early childhood is the ability to categorize geometrical shapes [51]. To have a more nuanced understanding of the effect of the intervention, we will assess the effect on both types of geometric knowledge: intuitive and academic.

The second aim of this study was to test the resilience of the intervention effect six months later, once the children were in the middle of first grade. An important debate about the long-term effects of early intervention programs has arisen in the last 10 years. If the children’s math performance in the preschool is associated with their math performance several years later, then early intervention programs would be expected to have lasting effects on children’s math achievement. However, recent research has called this conclusion into question [56–60]. Studies testing the medium and long-term effects of early math interventions show little [58] or no effect [13]. Nonetheless, studies that include a delayed posttest some weeks after the end of the learning phase are often successful in finding evidence of an intervention effect [1,61]. Accordingly, in this study the effect of the intervention was measured at two time points, immediately after learning and six months later to assess whether the gains would persist over time.

The results of this study are particularly relevant for Colombian population as there is a persistent gap between Colombian children and children in other countries in the mathematics achievement. Colombian children tend to present a broad range of difficulties learning mathematics throughout the formal school years and traditionally perform worse than their peers from other countries on international assessments. For instance, the 2018 Program for International Student Assessment (PISA) report from the Organization for Economic Cooperation and Development (OECD) shows that 15-year-old Colombian students score low (m = 391) on the Mathematics test, below the average for participating OECD countries (m = 489). Therefore, it is important to promote the development and improvement of early childhood skills through innovative teaching practices that have an impact on later mathematics achievement.

It is worth mentioning that as this study was carried out during the COVID-19 pandemic, several restrictions on the intervention were established (see the Methods section for more details). Particularly, the current intervention used a blended modality, with both online and in-person sessions in a similar proportion. Most interventions in previous studies were delivered in-person, either through computer-based materials [27,31] or through physical materials [2,13]. This is the first study to measure the effects of a 3-factor field intervention through virtual platforms and activities delivered face-to-face at the participants’ schools.

## Method

### Design and participants

This study implemented a quasi-experimental pre-test and post-test research design with an experimental group that received the intervention and a business-as-usual control group [62] The study took place in 18 kindergarten classrooms in six urban public schools serving children from ethnically-diverse low- and middle-income families. The participating schools were selected by convenience and were located in two of the largest Colombian cities: Cali and Bogotá. All participants spoke Spanish as their first language. The ethical approval of the study was obtained from the Institutional Review Committee for Ethics in Research of the Pontificia Universidad Javeriana. During recruitment, information letters and consent forms were sent home with each student through the group teacher (*N* = 360 children). A total of 192 children (53,3%) returned the forms with written consent and were tested in the pretest. The final sample who remained until the first posttest of the study was composed of 117 children aged 5 to 6 years (Mage = 67.1 months; SD = 9.1 months), including a similar number of girls (55) and boys (62). From this sample, 72 children participated in Bogotá and 45 in Cali. In each of the six participating schools, half of the participants were semi-randomly assigned either to the intervention group (n = 55) or the control group (n = 62). The high attrition rate resulted from children dropping out of school because of COVID-19 restrictions (n = 35), children moving out of the city (n = 5), some families opting out of the posttest phase of the study (n = 15) and parental interference during the pretest or the posttest (n = 20). Additionally, only 96 children participated in the working memory tests carried out after posttest 1. At posttest 2, another group of children dropped out of school (n = 25) or parents opted not to keep participating in the study (n = 8), for a final sample at posttest 2 of 84 children (Ncontrol = 40 and Nintervention = 44).

### Measures

Pretest measures were administered during lockdown where personal contact with children was restricted. As a consequence, all instruments were adapted to measure math knowledge through online sessions. Posttest 1 and 2 were administered in the same way with the same material. The instruments and changes are presented below for both numerical and geometric knowledge.

#### Number

Numerical knowledge was evaluated through items taken from the Tema-3 test and the Tedi-Math test. For the Tema-3, a total of 60 items were adapted for an online presentation. The Tema-3 items are divided into two categories: formal and informal. A total of 32 items from the informal group and 28 items from the formal group were selected. The unselected items (12) were not found suitable for online presentation, as they required the use of manipulatives. The selected items were representative of number knowledge across the age range used in the original version of the test, from 3 to 8 years of age. Informal category items assessed skills such as counting, number comparison, informal calculation, and informal number concepts. Items from the formal category assessed skills such as number transcoding (e.g., reading and writing Arabic numerals), mastery of number facts, calculation skills and number concepts. We administered the test in the standardized manner: the first item administered was determined by the child’s age, after which we administered item by item in the same sequence until the child incorrectly answered five items in a row. Items before the starting point were administered only if the child was unsuccessful on five consecutive items. No feedback was provided on any item.

From the Tedi-Math test we used only subscale 5 (operations), to assess arithmetic knowledge. We selected those items that were suitable for children in the age range of our sample; items assessing multiplication, division and missing-term problems were not included. A total of 23 items were administered, all of which were easily adaptable for an online presentation. Six items (5.A.) assessed children’s arithmetic skills with supporting images, and five items (5.B.1.) assessed arithmetic skills with Arabic notation and 12 items (5.C.) assessed performance in simple word problems. The children completed the test in the same order starting with the first item in the 5.A. scale. Testing stopped after five consecutive incorrect answers in the 5.C. scale. No feedback was provided on any item.

#### Geometry

**Fig 1:**
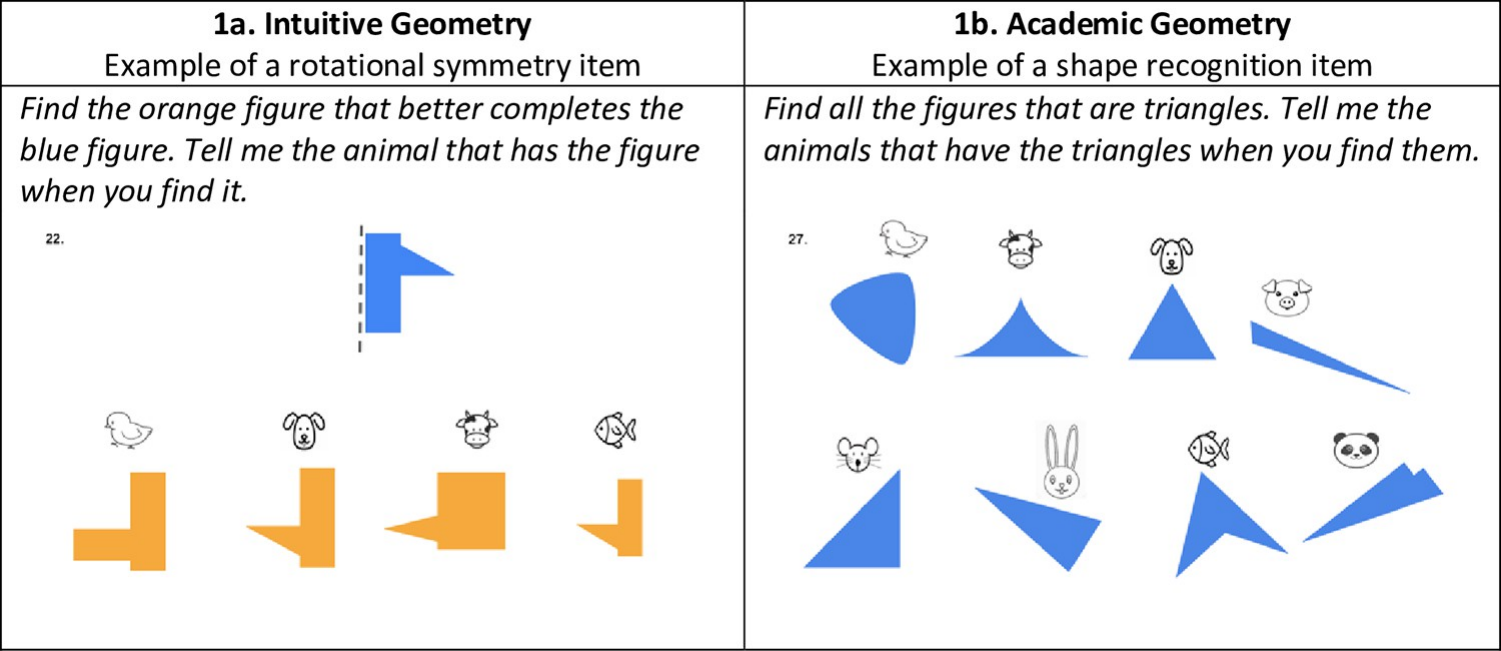
Examples of the type of items in the Geometry task.

The Geometry task was particularly designed for this study for an online presentation. The task was based on statutory learning requirements for preschool and first grade students in Colombia. The task included two sections, one encompassing 18 items to assess Intuitive geometry and one encompassing 12 items to assess Academic geometry, for a total of 30 items. The *Intuitive geometry* section included 6 items of congruence from translation, 4 items of congruence from rotation, 4 items of bilateral symmetry and 4 items of rotational symmetry (Fig 1A). The *Academic geometry* section included six items of shape naming and six items of shape identification (Fig 1B). Shape identification items were adapted from the tasks reported previously [51]. Based on the two sections of the Geometry task we calculated three scores: Intuitive geometry, Academic geometry and the Total score of the Geometry task.

Because many preschool children do not know letters and the geometry task was presented online, we used animal shapes to name the response options within each item. The geometry task started with a familiarization slide displaying eight animal figures and we asked the children to name each one. After that, a task was presented in which children were asked to match two non-geometric figures by naming the animal figure just above each of the four options shown. All the children in our sample named the animal figures and solved the familiarization task without any problems. In all the items of the Geometry task, the children had to mark their answer by providing the name of the animal figure above each of the different options. One-choice items from 1 to 24 were awarded one point if the response was correct and zero points if the response was incorrect. The last six multiple-choice shape recognition items were awarded 2 points for each correct response selected and minus 1 point for each incorrect response selected. The maximum score for the geometry task was 66. No feedback was provided on any item.

#### Working memory

Two working memory tests were administered: verbal and visuospatial working memory. Both tests were administered in a single in-person session once posttest 1 was completed. These measures were included as covariates in the statistical model as previous studies have demonstrated that working memory is associated with children’s performance on mathematical tests [61,63].

The *digit span backward task* was adopted from WISC-III and used to assess verbal working memory. In this task, the children were asked to repeat aloud a list of numbers in reverse order. Numbers were presented orally at a rate of one digit per second. The task started with sequences of a two-digit length and progressed to sequences of an eight-digit length. For each digit length, two sequences were presented. Testing stopped after two sequences of the same length were incorrectly repeated. The final score was the total number of correct trials.

A computerized version of the *Corsi Block-Tapping task* was also administered. The children were presented with nine blue boxes on a black screen background. In each trial, a number of these boxes light up in a prefixed sequence, constant across participants. After that, the children were asked to click on the boxes in the same order they were lit. The sequence length starts with two boxes and increases up to nine boxes. For each length two sequences were presented and testing stopped after two trials of the same length were incorrectly repeated. A 14” laptop was used to present this task. The final score was the total number of correct trials.

### Procedure

Before administering the pretest, each research assistant received training over a two-week period. The training consisted of joint study sessions of the test material and several online pilot tests, followed by a joint evaluation session. This cycle of study, piloting and evaluation was repeated until each research assistant achieved good performance. All pre-and-posttest measures were administered individually by the research assistants in two sessions of approximately 35 minutes each. The number tests and the Geometry task were administered through the Zoom platform. In one of the sessions, only the Tema-3 test was administered and in the other session, both the Tedi-Math test and the Geometry task were administered. The order of application was counterbalanced across participants. Parents were asked to either leave their children alone or to be quiet during the application to avoid interference. The pretest was administered within a 3-week window in May 2021 and the intervention started in August 2021. Although when posttest 1 and 2 were administered, children were allowed to attend school in person, we decided to administer the test online again to hold testing conditions constant. The posttests 1 and 2 were administered in a 2-week window in November 2021 and May 2022, respectively. The posttest 1 was administered one week after the intervention program ended. The working memory tests were administered once posttest 1 was finished.

### Intervention program

The researchers designed and scripted all the activities of the intervention program. Before the intervention sessions, the researchers trained nine research assistants to implement the activities with the children, faithfully following the task scripts. Research assistants were asked to practice task implementation, and emergent questions were discussed in a weekly research seminar.

The intervention program was implemented with the regular group classrooms in the experimental condition during the second semester of the school year from mid- August through early November 2021. The intervention program consisted of a set of activities organized around the three critical factors: symbolic number knowledge, spatial reasoning and mapping processes. The intervention program lasted 11 weeks, with three 40-minute sessions per week, one session for each factor. In total, the intervention program included 33 sessions, 11 for each factor. Weekly, the factors were rotated between the three days of implementation. Within each 40-minute session each research assistant implemented two activities of the same factor.

Because of the COVID-19 pandemic, several restrictions were introduced nationwide by the Colombian government during the second semester of 2021 in both public and private schools, which influenced the decisions made to implement the intervention of this study. First, during this semester, schools were utilizing a blended instruction model that combined both in-person and online teaching strategies; therefore, the implementation of the intervention activities took place under this model, alternating between both modalities. For each factor, five or six of the sessions were implemented online through digital platforms, and the other five or six sessions were implemented in-person in the usual classroom assigned to each group. Second, in-person sessions were restricted to small groups of children due to indoor capacity restrictions in schools as a safety measure. As the school teachers divided their classrooms into similar small groups, the number of students was mostly the same during the online sessions and during the in-person sessions. Third, children’s interactions in the classroom were limited to maintaining physical distancing. Accordingly, the intervention activities and the rules for the use of the materials were designed taking these restrictions into account.

#### Activities and pedagogical principles

The researchers of this study designed all the activities following previously defined pedagogical principles grounded on prior theory and research-based evidence. First, for each factor, activities were designed as part of broad learning environments that allowed participants to place each problem or task within a meaningful context. Each learning environment encompassed various activities and each activity included a set of tasks with a similar goal structure; mostly, each task included one example and ten items. During the intervention, these tasks were implemented across various sessions and their cognitive complexity increased within the sessions and throughout the sessions in order to promote improvement of the abilities and ways of reasoning. Table 1 presents the list of activities for each factor, the skills they attempt to develop, the modality in which they were implemented and the criteria utilized to increase the cognitive demand levels.

**Table 1:** Characteristics of the intervention activities.

Factor	Activity	Ability	Modality	Cognitive demand criteria
**Spatial reasoning**	*Castle of doors*	Mental rotation	Virtual challenge	• Key shape • Rotation degree • Direct/mediated interaction
	*Gardens of treasures*	Spatial scaling with use of maps	In-person challenge	• Presence or absence of reference point • Triangle configuration type • Sides on axes or not on axes • Real space rotation or no rotation
	*House of shapes*	Sensitivity to geometrical shapes	Virtual challenge	• Number of sides • Defining properties
	*Museum of art*	Sensitivity to geometrical relationships	In-person challenge	• Configuration type and orientation • Number of configuration figures • Number of positions moved
**Mapping processes**	*Number roulette*	Implicit number line representations	In-person manipulatives-based game	• Number range (up to 20) • Distance between quantities
	*Number line train*	Explicit number line representations	Virtual game	• Number range (up to 20) • -Distance between quantities
	*The magic world*	Number estimation	Virtual game	• Number range (up to 20) • Distance between quantities
	*Suns and stars*	Number estimation	In-person manipulatives-based game	• Number range (up to 20) • Distance between quantities
**Symbolic number knowledge**	*Kitchen inventory*	Basic counting strategies	In-person problem-solving activity	• Number range (up to 20)
	*Toy room inventory*	Finger counting on strategies	Virtual problem-solving activities	• Number range (up to 20) • Number of visible toys
	*Math card games*	Number identification	In-person manipulatives-based games	• Number range (up to 20) • Quantity representation format
	*The biggest number*	Number comparison	In-person manipulatives-based games	• Number range (up to 20) • Distance between numbers
	*Story problems*	Aditive composition	Virtual problem-solving activity	• Number range (up to 20) • Additive composition problem type (combination or transformation)
	*Find the partners*	Additive composition	In-person manipulatives-based games	• Number range (up to 20) • Abstractness of the diagrammatic tools
	*Math art pieces*	Additive composition	In-person manipulatives-based games	• Number range (up to 20) • Abstractness of the diagrammatic tools

All learning environments were created, designed, and implemented using a game-based learning pedagogical model. Consistent with this approach, activities were playful, developmentally appropriate for preschool instruction, and have a challenging goal to foster student engagement and conceptual understanding [3,4,51]. In what follows, these characteristics will be described for the group of activities within each factor and will be illustrated through one activity.

**Fig 2:**
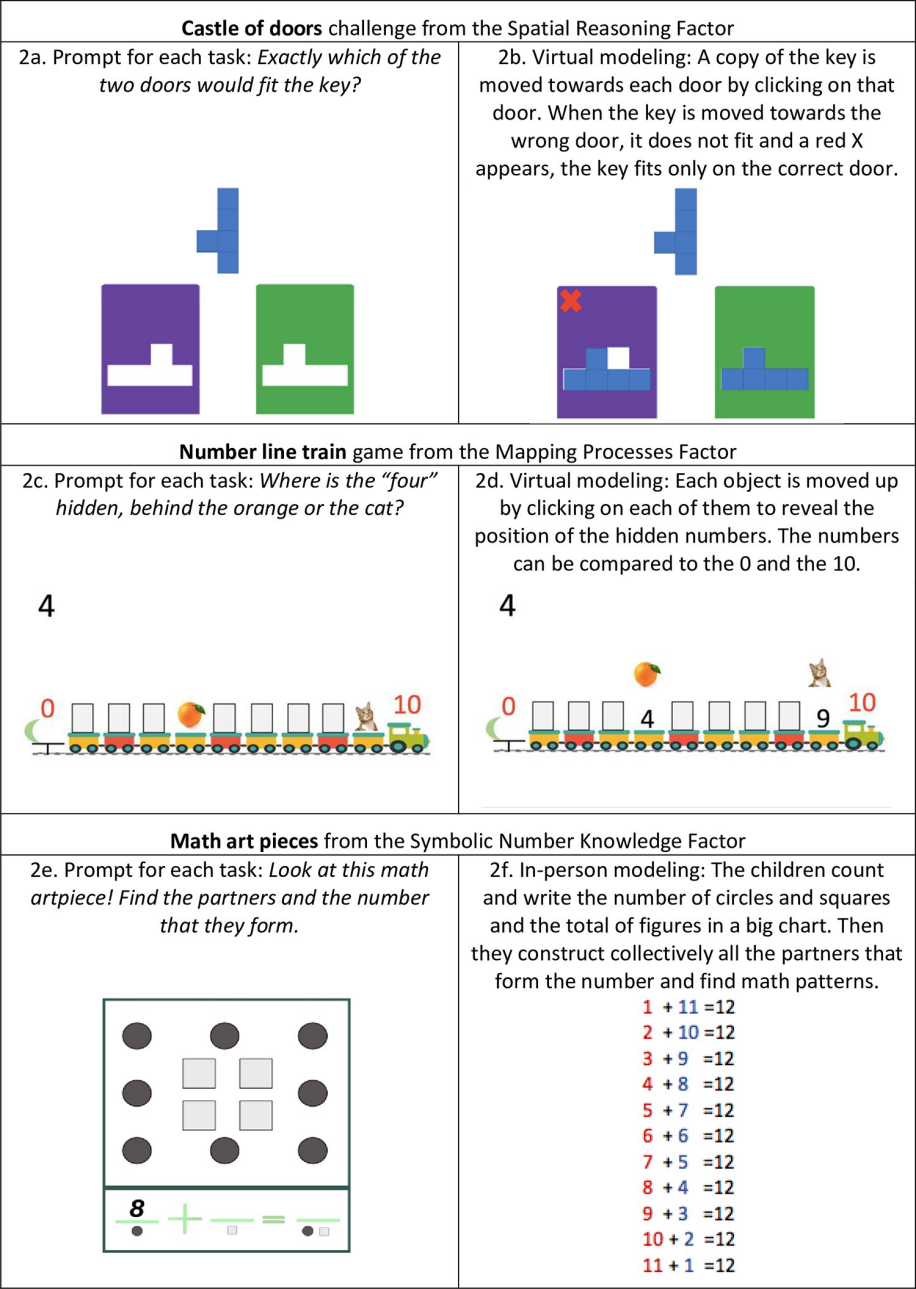
Selected examples of intervention activities from the three factors.

Four activities in the spatial reasoning factor were designed as challenges within a learning environment called *Iris’s Journey*. This microworld tells the story of Iris, a girl who traveled to *Spaceland*, the fantastic world of the forms and the space where a naughty wizard does mischief. Iris found four challenges and must solve them one by one to continue her journey. During activity implementation, children were presented with each challenge and asked to help Iris to solve it to encourage agency in problem solving. The four challenges were designed to improve four spatial abilities, namely mental rotation, spatial scaling, sensitivity to geometrical properties and sensitivity to geometric relationships. Skills such as mental rotation and spatial scaling were emphasized; therefore, the tasks addressing these processes were implemented over approximately seven sessions. Tasks related to sensitivity to geometric properties were addressed in five sessions and sensitivity to geometric relationships was addressed in only three sessions. Fig 2A presents a selected example of the *Castle of doors* virtual challenge designed to promote mental rotation skills. In this challenge, the children were told that Iris found a Castle with keys and doors and that she must go through several of these doors in order to get out of the Castle. But the naughty wizard put just one key in front of two doors, so Iris wondered which door each key would open. For each task item, the children were shown a slide with one target key at the top and two doors below, each with the silhouette of a key representing a hole. The children were asked to help Iris to figure out which of the two doors the key would fit exactly into. The children should select the door that has the silhouette with the same shape and size as the target key but rotated and must avoid the door with the mirror image of the key silhouette. Virtual modeling to verify the answer consisted of clicking on the door selected and a copy of the key moved to that door and turned it (see Fig 2B). When the key was on the wrong door the key would not fit and a red X appears. Then, a click on the second door made the copy of the key to move towards that door and rotate over that door. In the correct door the key fits exactly in the hole. An in-person version of the task included handouts with the keys and doors figures, and colorful dynamic keys to check children’s decisions. Cognitive complexity increased according to the degree of rotation, the shape of the key, and the type of interaction with the key–direct or mediated by the assistant.

Four activities in the mapping processes factor were designed as computer-based games or manipulatives-based games. Each game was a learning environment unto itself. The four games were designed to improve the number estimation and number line representation skills and both abilities were equally emphasized. Therefore, each activity was implemented in approximately six sessions. Fig 2C presents a selected example of the virtual *Number line train* game designed to promote explicit representations of the number line. In this game, the children were shown a slide with a train that has a number 0 on the train tile at the right-hand side, numbers 1 to 9 on each of the nine cars, and a number 10 on the locomotive at the left side. The children were told that the train travels to another city but at night the driver closes the windows; simultaneously the children were shown the same train with the numbers 0 and 10, but the numbers 1 to 9 were hidden on each carriage behind white windows. For each turn, the children were shown the train with the 0 and the 10 visible, and the numbers 1 to 9 hidden behind the windows except for two numbers that are hidden behind objects in the cars (e.g., an orange on the car where the 4 was hidden and a cat on the wagon where the 9 was hidden, see Fig 2D). The children were asked to say where one of the two numbers was hidden (e.g., 4) so that they could figure out the correct position of the target number (e.g., the four is behind the orange). Virtual modeling to check the answer consisted of clicking on the two objects, so that the windows moved up to make the numbers visible (see Fig 2D). The children were then asked to compare the positions of the two numbers to the positions of 0 and 10. Cognitive complexity increased according to the range of numbers (up to 20) and how close or far the numbers where.

Seven activities in the symbolic number knowledge factor were designed within two learning environments. First, *Pedrito’s inventory* included varied puzzles and problem-solving activities inspired by daily life to foster four abilities: number identification, number comparison, additive composition and counting strategies (basic and advanced). Second, the *Games of the Partners* included a variety of playful activities designed to develop additive composition skills. In the symbolic number knowledge factor, abilities such as number identification, number comparison and additive composition were emphasized and the tasks addressing these processes were approximately implemented across seven sessions, while counting abilities were targeted in just three sessions. Fig 2E presents a selected example of the in-person manipulative-based game *Math Art Pieces* designed to promote additive composition strategies using math art. In this game, the children were presented with mathematical art pieces showing aesthetically symmetrical configurations of circles and squares. The children were asked to interpret the artwork by finding all the partners that can create a number (e.g. 12). Therefore, all the artworks showed different symmetrical configurations with varying amounts of circles and squares but all made up the same number (e.g. 1 square and 11 circles, 2 squares and 10 circles, 3 squares and 9 circles, and so on). The children were asked to write the Arabig numeral corresponding to each partner and the largest number they make, using a plus (**+)** sign and an equal (**=)** sign depicted below each math artwork. The modeling consisted of using 1x1 m. charts with examples of partners that form the number (e.g. 12). At the end, the children were asked to collectively built the list of all the partners that form the numbers and visualize the different mathematical patterns, for instance for the number 12: 1+11 = 12, 2+10 = 12; 3+9 = 12, and so on. They were then encouraged to reflect on the increasing pattern of the first addend and the decreasing pattern of the second addend and on how the different partners form the same number. Cognitive complexity increased according to the range of numbers (up to 20) and the abstraction of the diagrammatic configurations of the figures.

Regarding the pedagogical principles for instruction tools, *symbolic number knowledge* and *mapping process* activities include varied visual mathematical representations of numbers and quantities that strengthen the connections among them [18]. These activities also included diagrams and manipulatives that organize such representations into intended structures that resemble the mathematical structures of concepts and processes, for instance, linear board games [3,4] and the cardinality chart [18]. Similarly, spatial activities included varied visual representations and manipulatives of forms and spatial relationships [8,51,52] as well as dynamism [64,65]. Various activities promoted the use of different sensory modalities so that participants could use their senses, hand gestures and body movements to grasp the concepts [65,66].

Regarding the pedagogical principles for adult scaffolding, all the activities included four types of structured feedback that were introduced in each task by the research assistants under the same conditions and were sized according to the children’s needs: a) *Motivation*, to encourage a child who made a mistake to continue looking for other solutions; b) *Collaboration*, to ask a partner to help a child who made a mistake; c) *Questions about explanations and justifications*, to ask a child to explain how she manage to find a solution or justify why she thinks the solution is good or bad; and, d) *Modeling*, to verify or demonstrate an answer, a solution or a strategy through situated action or the use of dynamic tools both digital or physical (see Fig 2B, 2D and 2F for examples of modeling utilized within virtual and in-person activities). These feedback strategies were intended to foster collaborative learning and conceptual understanding. Particularly, the modeling strategies where focused on concept definitions, properties or relationships harnessing the mathematical, interactive, and communicational affordances of these dynamic technologies or physical artifacts.

Finally, although we did not systematically record the activities followed by teachers in the control group, we do know that they follow a traditional curriculum, which emphasizes counting activities from 1 to 10 and prototypical shape identification tasks. The time spend in these activities was approximately equivalent to the time spend in the intervention program.

## Results

### Performance at pretest and bivariate correlations

The descriptive information of the performance in each of the tasks, both for the control group and the intervention group, is shown in Table 2. No statistically significant differences were found in any measure (all ps > .05). The descriptive statistics show a possible ceiling effect in the Intuitive Geometry items at the pretest (83% of achievement for the intervention group), suggesting that Congruence and Symmetry items were generally easy for children in our sample. Furthermore, Pearson’s correlations indicated a medium positive association between the geometry test and all other number scores (Table 3). These results highlight that knowledge of geometry and number, although independent of each other, share some variance. Likewise, as expected, all number scores showed a high positive association. In particular, the formal and informal scores taken from the Tema-3 showed a very high correlation. Additionally, verbal working memory had a medium positive association with both formal and informal scores of the Tema-3 on Posttest 1. Neither verbal working memory nor visuospatial working memory showed significant associations with geometry scores. In verbal working memory, the children had a mean span of 2,03 digits (SD = 1,18), and in visuospatial working memory a mean score of 3,47 (SD = 1,98). Finally, no significant differences were found across Sex and City on any measure.

**Table 2:** Means and standard deviation of math tests.

		Pretest	Posttest 1	Posttest 2
**Geometry Test**	Control	32.35 (6.84)	33.16 (5.09)	31.74 (6.17)
	Intervention	31.56 (5.59)	35.61 (5.21)	34.86 (5.19)
Intuitive Geometry	Control	14.02 (2.6)	14.6 (2.87)	14.33 (3.65)
	Intervention	14.96 (2.45)	15.55 (2.52)	15.74 (2.15)
Academic Geometry	Control	18.56 (4.27)	18.56 (3.1)	17.41 (4.51)
	Intervention	16.6 (5.15)	20.06 (4.1)	19.11 (4.27)
**Tema-3 Test**	Control	14.73 (6.88)	21.94 (6.2)	25.89 (5.59)
	Intervention	16.08 (8.21)	25.35 (7.9)	26.58 (7.1)
Tema-3 Test Formal	Control	2.65 (1.87)	4.28 (1.99)	6.83 (2.77)
	Intervention	2.94 (2.2)	5.37 (2.6)	7.2 (2.48)
Tema-3 Test Informal	Control	12.08 (5.24)	17.66 (4.49)	18.38 (3.68)
	Intervention	12.92 (6)	19.79 (5.6)	19.36 (4.56)
**Tedi-Math Test**	Control	7.02 (3.6)	11.52 (4.14)	13.47 (3.76)
	Intervention	7.38 (4.54)	12.24 (4.4)	13.87 (4)

**Table 3:** Bivariate correlations between tasks at Posttest 1.

	Mathematical Tests				Working Memory Tests	
	1	2	3	4	5	6
**Geometry Test**						
**Tema-3 Test Formal**	0.31**					
**Tema-3 Test Informal**	0.31**	0.83**				
**Tedi-Math**	0.36**	0.56**	0.67**			
**Viso-spatial WM**	0.14	0.19	0.19	0.27**		
**Verbal WM**	0.19	0.39**	0.49**	0.2*	0.12	

* p < .05

** p < .01.

### Performance at posttest 1

ANCOVAs were used to investigate the effects of the intervention on children’s geometry and numerical performance. Preliminary analyses found no effect of Sex and City; therefore, these variables were collapsed in further analyses. Pretest scores were included as a covariate in the models. This analysis yielded a statistically significant difference between the control and intervention groups in the Tema-3 test, with higher scores in the latter group, *F*(1, 114) = 11.6, p < .01, η_p_^2^ = .09 (see Table 2). Further analyses were performed to investigate the separate effect on both formal and informal mathematics. ANCOVAs showed significant differences between the control and intervention group in the formal math score, *F*(1, 114) = 6.9, p = .01, η_p_^2^ = .06, and the Informal math score, *F*(1, 114) = 10,6, p < .01, η_p_^2^ = .08. However, when we compared the children’s performance on the subtest of the Tedi-Math there was not a reliable intervention effect, with similar performance in both groups, *F*(1, 112) = 2.9, p = .09, η_p_^2^ = .02. This lack of intervention effect was maintained across all three Tedi-Math subtests (all ps > .1).

A new set of statistical analyses was conducted to control for the children’s working memory skills. The above analyses were repeated with a smaller sample size (see the participants section) including verbal and visuospatial working memory as covariates in the models. ANCOVAs show the same prior pattern of results with significant differences in all measures (all ps < .01), except Tedi-Math (p = .34). Importantly, mixed ANOVAs were run in parallel to these analyses with Test (Pre–Post) as a within factor and Condition (Control–Intervention) as a between factor. Comparable results were obtained for all outcomes.

In the case of geometry, the ANCOVA revealed that the intervention group differed significantly from the control group, with better performance in the former, *F*(1, 114) = 5.8, p = .018, η_p_^2^ = .05. The ANCOVA revealed a significant intervention effect on the Academic Geometry tasks, *F*(1, 114) = 4,39, p = .038, η_p_^2^ = .04, with better scores for the intervention group than for the control group. However, there was no significant intervention effect for the Intuitive Geometry tasks, *F*(1, 114) = 2.6, p = .1, η_p_^2^ = .02. This lack of intervention effect could be the result of the possible ceiling effect found in the pretest. These analyses were repeated including verbal and visuospatial working memory as covariates, showing the same pattern of results (p = .02 for Academic and p = .12 for Intuitive). As before, mixed ANOVAs were run in parallel and comparable results were obtained for all outcomes.

To gain a deeper understanding of individual differences across pre and posttest measures, we calculated the difference between posttest 1 and the pretest for both the Tema-3 test and the Geometry Test. Then, we correlated this pre-post difference with the pretest scores for both the control and the intervention groups. The results for the Tema-3 test show a significant negative correlation for the intervention group (r = - 0,32, p = .015), but not for the control group (r = - 0,23, p = .062). These results indicate that children with low pretest scores tend to improve more throughout the study, but this is particularly evident when children participate in the intervention. Similarly, the results for the Geometry Test show a high significant negative correlation for both the Intervention Group (r = - 0,65, p < .01) and the Control Group (r = - 0,66, p < .01), indicating that overall, children with low scores at the pretest improved more throughout the study, regardless of their condition.

### Performance at posttest 2

To test for differences between the control and the intervention groups 6 months after finishing the intervention, a new set of ANCOVAs was performed. As before, the analyses found no effect of Sex and City. Pretest scores, verbal and visuospatial working memory were included as covariates in the models. Unlike posttest 1, there were no significant intervention effects on any of the numerical tests (all ps < .1). However, the effect of the intervention was still present in the geometry test, *F*(1, 79) = 9.6, p < .01, η_p_^2^ = .1, which resulted from a better children’s performance in the Intervention group than in the control group (see Table 1). Significant changes were found only on the Academic geometry tasks, *F*(1, 79) = 5.82, p = .018, η_p_^2^ = .07. These results were corroborated through mixed ANOVAs.

To analyze the changes in the children’s performance across the three time points, we conducted a series of repeated measures one-way ANOVAs for both the control and the intervention group. For both numerical measures, Tema-3 and Tedi-Math, the analysis yielded a statistically significant effect (all ps < .01). Planned t-test comparisons, Bonferroni corrected, between Pretest–Postest 1 and Postest 1—Postest 2, revealed significant differences (al ps < .01). For the geometry task, the one-way ANOVA yielded a statistically significant effect for the Intervention group, *F*(2, 90) = 9,35, p < .01, but not for the control group, *F*(2, 98) = 0,54, p = .58. Planned t-test comparisons for the intervention group, Bonferroni corrected, revealed a significant difference between the pretest and posttest 1, *t*(55) = -3,6, p < .01, but not for the comparison of posttest 1 and posttest 2, *t*(45) = 0,72, p = .47.

## Discussion

The current study investigated the short- and medium- term effects of a field math intervention program for preschool-aged children. All the tasks designed for this study were based on three developmental factors that have been associated with early children’s math performance: symbolic number knowledge, spatial reasoning, and mapping processes. Due to COVID-19 related restrictions, the intervention was implemented through a blended model turning between both online and in-person sessions. The effect of the intervention was tested through two different adapted number tests, Tema-3 and a subtest of the Tedi-Math, and a geometry task. Overall, the 11-week intervention was successful in showing larger changes in math skills for the intervention group than for the control group. This result demonstrates the importance of adapting well-known developmental factors studied in lab settings to more contextualized school settings. Most of the activities were game-based and challenging for children. Online environments were also appropriate for fostering some number, spatial and mapping skills requiring computer-based feedback and modeling (e.g., mental rotation, strategic counting, number line) and time pressure (e.g., number estimation). This study adds evidence to the growing literature showing the importance of developing early interventions to promote children’s math abilities [1,12,20,34,61].

In the current research, we also measure the effects of the intervention on various types of mathematical knowledge. The results of the Tema-3 test revealed that the intervention was effective for both formal and informal math knowledge. However, the results of the Tedi-math test did not show a significant effect of the intervention in any of the three subtests. We believe that this difference in the results is revealing. Unlike the Tema-3 test, the items of the Tedi-math are mainly focused on formal and informal arithmetic skills. Although the Tema-3 has some items to assess arithmetic skills, it explores a much wider variety of math skills. Therefore, the intervention had a rather weak effect on children’s arithmetic skills. One possibility to account for this divergent pattern of results is that the intervention program had an effect mainly on basic formal and informal math skills. This type of limited effect of early interventions has been shown in prior research. For instance, a study [2] showed that an intervention on a broad variety of spatial abilities improved kindergartners’ performance on a symbolic number comparison test, but not on a number knowledge test, measuring numerical calculation skills. Additionally, some studies on spatial reasoning skills such as mental rotation and scaling have shown a correlation between these skills and arithmetic performance that is already apparent in preschoolers [8]. However, most intervention studies with mental rotation and scaling on arithmetic performance have shown positive findings only for first grade children or older [12,27,35]. Therefore, these studies suggest that a wider effect on children’s math skills may require a longer and sustained intervention beyond the preschool years.

The intervention program was also effective in improving children’s geometry skills. A previous study [12] showed that an intervention on spatial reasoning improved the 8-year-olds’ performance on a geometry test. However, the current study expands these results by showing the effectiveness of a 3-factor field intervention in preschoolers. The analyses indicate that this effect was mainly the result of a better performance on Academic geometry items. One possibility is that the improvement in mental rotation, spatial scaling and sensitivity to geometric properties led the children to better analyze the defining geometric attributes and properties of 2-D figures, even when they are rotated or have varied sizes or have non-prototypical forms [51]. On the other hand, the tasks targeting sensitivity to geometrical relationships among shapes, which are more related to the Intuitive geometry items such as those evaluating congruence and symmetry, were less emphasized in the intervention. Perhaps this aspect influenced children’s non-significant gains in the Intuitive geometry items. However, the possible ceiling effect on the Intuitive geometry items makes it difficult to assess the effectiveness of the intervention on more basic geometric knowledge. For instance, it is an open question whether training on mental transformation skills such as mental rotation and spatial scaling may lead young children to better performance on intuitive geometry tasks with a higher rotational demand.

Additionally, correlational results also indicate that for numerical knowledge the intervention had a greater effect compared to the control condition for those children with low performance on the pretest. This is in line with previous studies showing successful intervention programs for children at risk of mathematical learning difficulties [1,3,4,61,67]. Many researchers have argued that specific characteristics of the training activities may contribute to helping low-income children at risk to improve their math abilities during their intervention programs. First, implementing materials that embed the structure of the mathematical concepts and processes for interventions in symbolic number knowledge and mapping processes may promote conceptual understanding. The activity materials designed for this study draw on this premise and include visual and diagrammatic tools that represent the mathematical structure of concepts and processes; for example, the number roulette includes a linear board game that facilitated implicit representations of the number line [3,4], and the cardinality chart represented the relationships between Arabic numbers and the corresponding increasing quantities [1,18]. Second, previous studies with elementary school children have shown that intervention programs on mental rotation implementing *explicit instruction* may influence the significant effects on mathematical performance [12,27]. This study utilized explicit instruction and structured feedback sized to the children’s needs that could contribute to their gains during the program. Third, a recent meta-analysis demonstrated that spatial reasoning training based on concrete materials produced larger gains in mathematical knowledge than computer-based training [22]. The present study utilized a blended online and in-person field implementation of the intervention. However, for both modalities we harnessed the affordances of varied pedagogical tools such as story contexts, visual and diagrammatic representations, digital and psychical dynamic objects, and structured manipulatives. It is possible that using these tools facilitated children’s modeling and explanation of their thinking [18,65]. Therefore, an early field intervention targeting number, spatial and mapping abilities and, drawing on pedagogical tools critical for student learning may contribute to reducing the achievement gap between low and high achievers.

The second aim of the current research was to test the resilience of the math intervention effect. The results show that 6 months after the end of the intervention program, the effect on number knowledge has faded and intervention and control groups had overall the same performance. This finding is largely in line with previous studies testing the medium- and long-term effects of early intervention programs [13,60]. Why is it the case that the effect of a preschool math intervention program fades over time? After all, several studies have demonstrated that preschoolers with better math skills perform better throughout elementary school [5,6]. One possibility is that the effect is weak and thus susceptible to fading in a regular educational setting [58]. Under this interpretation, the children’s improvement in this study may not be sufficiently consolidated and the new knowledge is forgotten over time. However, the children in both comparison groups improved their performance between Posttest 1 and 2, suggesting instead that children in the Control group “catch up” with their peers in the intervention group. Another possibility is that the newly acquired knowledge does not have a direct impact on the more complex math knowledge that children learn during elementary school. However, this would be at odds with studies showing that preschoolers with better basic math skills, such as counting and number comparison, have better arithmetic skills [5,9]. Either way, these findings suggest that early intervention programs need to be supplemented by ongoing educational support beyond the preschool years.

In contrast to number, geometry knowledge had a resilient intervention effect on posttest 2. This finding must be qualified by the fact that number and geometry follow different developmental trajectories. The control group displayed a steady increase in their numerical performance, but showed no change in geometric knowledge between posttest 1 and 2. Although present in the curriculum of our country, geometry is not the main focus of educational activities in preschool. Therefore, we believe that training in spatial reasoning boosted children’s geometry performance under a more stable knowledge background. This also adds evidence against the interpretation that the effect of the intervention on number was weak and knowledge forgotten over time. Much of the medium- and long-term effect of early intervention programs depends on the educational context of elementary school and its learning objectives.

### Limitations

The findings of the current study should be considered in light of several limitations. First, as a result of restrictions related to COVID-19, the intervention was carried out through a blended modality, which is difficult to replicate in normal circumstances. However, we also believe that for the same reason this study contributes to understanding the conditions under which the effectiveness of early math interventions is generalizable [68]. Several recent studies have shown the challenges teachers around the world faced during the pandemic when undertaking online learning activities, which in some cases were described as a “learning loss” [69–72]. The research findings presented in this article show that computer-mediated activities are at least feasible to implement in preschool with positive learning effects.

Second, due to the lockdown, we had to adapt the assessment instruments to test children in online sessions. Some items in the Tema-3 could not be tested in this scenario, and others were transformed. Therefore, it is impossible to use these results to compare the performance of the children in our sample to a normative scale. Additionally, associated with this testing modality, we had a high dropout rate as some parents interfered by prompting the children’s responses and were therefore discarded. Since the pre and posttest measures were collected in a less controlled environment than typical intervention studies, the results of this study should be interpreted with caution. Nevertheless, we believe that by holding the testing conditions constant throughout the study, the effect of the intervention can be detected.

Third, because we implemented an important number of different activities, we cannot be sure what caused the effect on the children’s math learning. We believe that all three critical factors–symbolic number, spatial reasoning, and mapping- contributed to some extent to improving the children’s performance. However, it is reasonable to think that each had an effect on different dimensions of math knowledge. For instance, it is expected that mapping abilities had an effect on formal math knowledge, but not on children’s counting skills. Whether or not this was the case is an open question. Similarly, we cannot prove the causal role in the children’s gains of each of the pedagogical principles used in the study. Future research should address the differential effects of using such a variety of tools and scaffolding on students’ mathematical performance.

## Conclusion

The current study provided evidence for the effectiveness of an early field intervention program on both number and geometry knowledge. All activities developed for this study were based on recent findings on children’s math development regarding number, space, and mapping processes, and were successfully implemented in school settings. However, the results for the medium-term effect were mixed, with null results for numerical knowledge and a robust intervention effect still present for geometry knowledge. Future studies should advance the implementation of this type of intervention program in regular school activities.

## References

[1] Dyson N, Jordan NC, Beliakoff A, Hassinger-Das B. A kindergarten number-sense intervention with contrasting practice conditions for low-achieving children. Journal for Research in Mathematics Education. 2015; 46(3):331–70. doi: 10.5951/jresematheduc.46.3.0331 26388651 PMC4572740

[2] Hawes Z, Moss J, Caswell B, Naqvi S, MacKinnon S. Enhancing children’s spatial and numerical skills through a dynamic spatial approach to early geometry instruction: Effects of a 32-week intervention. Cognition and Instruction. 2017; 35(3):236–64.

[3] Siegler RS, Ramani GB. Playing linear number board games—but not circular ones—improves low-income preschoolers’ numerical understanding. Journal of educational psychology. 2009; 101(3):545.

[4] Siegler RS, Ramani GB. Playing linear numerical board games promotes low‐income children’s numerical development. Developmental science. 2008; 11(5):655–61. doi: 10.1111/j.1467-7687.2008.00714.x 18801120

[5] Hirsch S, Lambert K, Coppens K, Moeller K. Basic numerical competences in large-scale assessment data: Structure and long-term relevance. Journal of Experimental Child Psychology. 2018; 167:32–48. doi: 10.1016/j.jecp.2017.09.015 29154029

[6] Jordan NC, Kaplan D, Ramineni C, Locuniak MN. Early math matters: kindergarten number competence and later mathematics outcomes. Developmental psychology. 2009; 45(3):850. doi: 10.1037/a0014939 19413436 PMC2782699

[7] Clements DH, Sarama J. Effects of a preschool mathematics curriculum: Summative research on the Building Blocks project. Journal for research in Mathematics Education. 2007; 38(2):136–63.

[8] Frick A. Spatial transformation abilities and their relation to later mathematics performance. Psychological Research. 2019; 83(7):1465–84. doi: 10.1007/s00426-018-1008-5 29637258

[9] Göbel SM, Watson SE, Lervåg A, Hulme C. Children’s arithmetic development: It is number knowledge, not the approximate number sense, that counts. Psychological science. 2014; 25(3):789–98. doi: 10.1177/0956797613516471 24482406

[10] Malone SA, Heron-Delaney M, Burgoyne K, Hulme C. Learning correspondences between magnitudes, symbols and words: Evidence for a triple code model of arithmetic development. Cognition. 2019; 187:1–9. doi: 10.1016/j.cognition.2018.11.016 30797098

[11] Gilligan KA, Flouri E, Farran EK. The contribution of spatial ability to mathematics achievement in middle childhood. Journal of experimental child psychology. 2017; 163:107–25. doi: 10.1016/j.jecp.2017.04.016 28753435

[12] Gilligan KA, Thomas MS, Farran EK. First demonstration of effective spatial training for near transfer to spatial performance and far transfer to a range of mathematics skills at 8 years. Developmental Science. 2020; 23(4):e12909. 31599470 10.1111/desc.12909 PMC7379338

[13] Dillon MR, Kannan H, Dean JT, Spelke ES, Duflo E. Cognitive science in the field: A preschool intervention durably enhances intuitive but not formal mathematics. Science. 2017; 357(6346):47–55. doi: 10.1126/science.aal4724 28684518

[14] Libertus ME, Forsman L, Adén U, Hellgren K. Deficits in approximate number system acuity and mathematical abilities in 6.5-year-old children born extremely preterm. Frontiers in Psychology. 2017; 8:1175. doi: 10.3389/fpsyg.2017.01175 28744252 PMC5504250

[15] LeFevre JA, Smith-Chant BL, Fast L, Skwarchuk SL, Sargla E, Arnup JS, Penner-Wilger M, Bisanz J, Kamawar D. What counts as knowing? The development of conceptual and procedural knowledge of counting from kindergarten through Grade 2. Journal of experimental child psychology. 2006; 93(4):285–303. doi: 10.1016/j.jecp.2005.11.002 16360166

[16] Stock P, Desoete A, Roeyers H. Predicting arithmetic abilities: The role of preparatory arithmetic markers and intelligence. Journal of Psychoeducational Assessment. 2009; 27(3):237–51.

[17] Martin RB, Cirino PT, Sharp C, Barnes M. Number and counting skills in kindergarten as predictors of grade 1 mathematical skills. Learning and individual differences. 2014; 34:12–23. doi: 10.1016/j.lindif.2014.05.006 25089081 PMC4116688

[18] Fuson KC. Relating math words, visual images, and math symbols for understanding and competence. International journal of disability, development and education. 2019; 66(2):119–32.

[19] Purpura DJ, Baroody AJ, Lonigan CJ. The transition from informal to formal mathematical knowledge: Mediation by numeral knowledge. Journal of Educational Psychology. 2013; 105(2):453.

[20] Bojorque G, Torbeyns J, Van Hoof J, Van Nijlen D, Verschaffel L. Effectiveness of the Building Blocks program for enhancing Ecuadorian kindergartners’ numerical competencies. Early Childhood Research Quarterly. 2018; 44:231–41.

[21] Uttal DH, Meadow NG, Tipton E, Hand LL, Alden AR, Warren C, et al. The malleability of spatial skills: a meta-analysis of training studies. Psychological bulletin. 2013; 139(2):352. doi: 10.1037/a0028446 22663761

[22] Gilligan-Lee KA, Hawes ZC, Mix KS. Spatial thinking as the missing piece in mathematics curricula. npj Science of Learning. 2022; 7(1):1–4. 35654949 10.1038/s41539-022-00128-9 PMC9163079

[23] Newcombe NS, Frick A. Early education for spatial intelligence: Why, what, and how. Mind, Brain, and Education. 2010; 4(3):102–11.

[24] Hawes ZC, Gilligan-Lee KA, Mix KS. Effects of spatial training on mathematics performance: A meta-analysis. Developmental Psychology. 2022; 58(1):112. doi: 10.1037/dev0001281 35073120

[25] Lourenco SF, Cheung CN, Aulet LS. Is visuospatial reasoning related to early mathematical development? A critical review. Heterogeneity of function in numerical cognition. 2018:177–210.

[26] Yang W, Liu H, Chen N, Xu P, Lin X. Is early spatial skills training effective? A meta-analysis. Frontiers in psychology. 2020; 11:1938. doi: 10.3389/fpsyg.2020.01938 32982829 PMC7485443

[27] Cheng YL, Mix KS. Spatial training improves children’s mathematics ability. Journal of cognition and development. 2014; 15(1):2–11.

[28] Verdine BN, Golinkoff RM, Hirsh-Pasek K, Newcombe NS. Finding the missing piece: Blocks, puzzles, and shapes fuel school readiness. Trends in Neuroscience and Education. 2014; 3(1):7–13.

[29] Möhring W, Newcombe NS, Frick A. The relation between spatial thinking and proportional reasoning in preschoolers. Journal of Experimental Child Psychology. 2015;132:213–20. doi: 10.1016/j.jecp.2015.01.005 25705050

[30] Cornu V, Schiltz C, Pazouki T, Martin R. Training early visuo-spatial abilities: A controlled classroom-based intervention study. Applied Developmental Science. 2019; 23(1):1–21.

[31] Hawes Z, Moss J, Caswell B, Poliszczuk D. Effects of mental rotation training on children’s spatial and mathematics performance: A randomized controlled study. Trends in Neuroscience and Education. 2015; 4(3):60–8.

[32] Lowrie T, Logan T, Ramful A. Visuospatial training improves elementary students’ mathematics performance. British Journal of Educational Psychology. 2017; 87(2):170–86. doi: 10.1111/bjep.12142 28097646

[33] Rodán A, Gimeno P, Elosúa MR, Montoro PR, Contreras MJ. Boys and girls gain in spatial, but not in mathematical ability after mental rotation training in primary education. Learning and Individual Differences. 2019; 70:1–1.

[34] Bower C, Zimmermann L, Verdine B, Toub TS, Islam S, Foster L, et al. Piecing together the role of a spatial assembly intervention in preschoolers’ spatial and mathematics learning: Influences of gesture, spatial language, and socioeconomic status. Developmental Psychology. 2020; 56(4):686. doi: 10.1037/dev0000899 32134293

[35] Cheung CN, Sung JY, Lourenco SF. Does training mental rotation transfer to gains in mathematical competence? Assessment of an at-home visuospatial intervention. Psychological Research. 2020; 84(7):2000–17. doi: 10.1007/s00426-019-01202-5 31144028

[36] Siegler RS. Magnitude knowledge: The common core of numerical development. Developmental science. 2016; 19(3):341–61. doi: 10.1111/desc.12395 27074723

[37] Chen Q, Li J. Association between individual differences in non-symbolic number acuity and math performance: A meta-analysis. Acta psychologica. 2014; 148:163–72. doi: 10.1016/j.actpsy.2014.01.016 24583622

[38] Halberda J, Mazzocco MM, Feigenson L. Individual differences in non-verbal number acuity correlate with maths achievement. Nature. 2008; 455(7213):665–8. doi: 10.1038/nature07246 18776888

[39] Schneider M, Beeres K, Coban L, Merz S, Susan Schmidt S, Stricker J, et al. Associations of non‐symbolic and symbolic numerical magnitude processing with mathematical competence: A meta‐analysis. Developmental science. 2017; 20(3):e12372. doi: 10.1111/desc.12372 26768176

[40] Brankaer C, Ghesquière P, De Smedt B. Numerical magnitude processing deficits in children with mathematical difficulties are independent of intelligence. Research in Developmental Disabilities. 2014; 35(11):2603–13. doi: 10.1016/j.ridd.2014.06.022 25036314

[41] Feldman A, Berger A. Development of the Mental Number Line Representation of Numbers 0–10 and Its Relationship to Mental Arithmetic. Brain Sciences. 2022; 12(3):335. doi: 10.3390/brainsci12030335 35326291 PMC8946762

[42] Kolkman ME, Kroesbergen EH, Leseman PP. Early numerical development and the role of non-symbolic and symbolic skills. Learning and instruction. 2013; 25:95–103.

[43] Siegler RS, Opfer JE. The development of numerical estimation: Evidence for multiple representations of numerical quantity. Psychological science. 2003; 14(3):237–50. doi: 10.1111/1467-9280.02438 12741747

[44] Lange AA, Brenneman K, Sareh N. Using number games to support mathematical learning in preschool and home environments. Early Education and Development. 2021; 32(3):459–79.

[45] Laski EV, Siegler RS. Learning from number board games: You learn what you encode. Developmental psychology. 2014; 50(3):853. doi: 10.1037/a0034321 24099546

[46] Maertens B, De Smedt B, Sasanguie D, Elen J, Reynvoet B. Enhancing arithmetic in pre-schoolers with comparison or number line estimation training: Does it matter? Learning and Instruction. 2016; 46:1–1.

[47] Dyson NI, Jordan NC, Glutting J. A number sense intervention for low-income kindergartners at risk for mathematics difficulties. Journal of learning disabilities. 2013; 46(2):166–81. doi: 10.1177/0022219411410233 21685346 PMC3566272

[48] Rousselle L, Noël MP. Basic numerical skills in children with mathematics learning disabilities: A comparison of symbolic vs non-symbolic number magnitude processing. Cognition. 2007; 102(3):361–95. doi: 10.1016/j.cognition.2006.01.005 16488405

[49] Namkung JM, Peng P, Lin X. The relation between mathematics anxiety and mathematics performance among school-aged students: A meta-analysis. Review of Educational Research. 2019; 89(3):459–96.

[50] Ünlü M, Ertekin E. A structural equation model for factors affecting eighth graders’ geometry achievement. Educational Sciences: Theory & Practice. 2017; 17(5).

[51] Clements DH, Sarama J. Learning and teaching early math: The learning trajectories approach. Routledge; 2014.

[52] Frick A, Newcombe NS. Getting the big picture: Development of spatial scaling abilities. Cognitive Development. 2012; 27(3):270–82.

[53] Giofrè D, Mammarella IC, Cornoldi C. The relationship among geometry, working memory, and intelligence in children. Journal of Experimental Child Psychology. 2014; 123:112–28. doi: 10.1016/j.jecp.2014.01.002 24709286

[54] Dehaene S, Izard V, Pica P, Spelke E. Core knowledge of geometry in an Amazonian indigene group. Science. 2006; 311(5759):381–4. doi: 10.1126/science.1121739 16424341

[55] Spelke E, Lee SA, Izard V. Beyond core knowledge: Natural geometry. Cognitive science. 2010; 34(5):863–84. doi: 10.1111/j.1551-6709.2010.01110.x 20625445 PMC2897178

[56] Bailey DH, Watts TW, Littlefield AK, Geary DC. State and trait effects on individual differences in children’s mathematical development. Psychological science. 2014; 25(11):2017–26. doi: 10.1177/0956797614547539 25231900 PMC4292922

[57] Clarke B, Doabler C, Smolkowski K, Kurtz Nelson E, Fien H, Baker SK, et al. Testing the immediate and long-term efficacy of a tier 2 kindergarten mathematics intervention. Journal of Research on Educational Effectiveness. 2016; 9(4):607–34.

[58] Clements DH, Sarama J, Wolfe CB, Spitler ME. Longitudinal evaluation of a scale-up model for teaching mathematics with trajectories and technologies: Persistence of effects in the third year. American Educational Research Journal. 2013; 50(4):812–50.

[59] Taylor E. Spending more of the school day in math class: Evidence from a regression discontinuity in middle school. Journal of Public Economics. 2014; 117:162–81.

[60] Watts TW, Duncan GJ, Clements DH, Sarama J. What is the long‐run impact of learning mathematics during preschool?. Child development. 2018; 89(2):539–55. doi: 10.1111/cdev.12713 28105650 PMC5519454

[61] Aunio P, Korhonen J, Ragpot L, Törmänen M, Henning E. An early numeracy intervention for first-graders at risk for mathematical learning difficulties. Early Childhood Research Quarterly. 2021; 55:252–62.

[62] Shaughnessy JJ, Zechmeister EB, Zechmeister JS. Research methods in psychology. McGraw-Hill; 2000.

[63] Geary DC, Hoard MK, Byrd-Craven J, DeSoto MC. Strategy choices in simple and complex addition: Contributions of working memory and counting knowledge for children with mathematical disability. Journal of experimental child psychology. 2004; 88(2):121–51. doi: 10.1016/j.jecp.2004.03.002 15157755

[64] Hegedus SJ, Otálora Y. Mathematical strategies and emergence of socially mediated metacognition within a multi-touch Dynamic Geometry Environment. Educational Studies in Mathematics. 2022:1–9.35034972

[65] Sinclair N, De Freitas E, Ferrara F. Virtual encounters: The murky and furtive world of mathematical inventiveness. ZDM. 2013; 45(2):239–52.

[66] Stull AT, Hegarty M, Cook SW, Markman AB, Morrison RG, Goldin-Meadow S, Holyoak KJ. Gesture in Thought. In The Oxford Handbook of Thinking and Reasoning 2012. Oxford University Press.

[67] Ramani GB, Siegler RS. Reducing the gap in numerical knowledge between low-and middle-income preschoolers. Journal of applied developmental Psychology. 2011; 32(3):146–59.

[68] Nosek BA, Errington TM. What is replication?. PLoS biology. 2020;18(3):e3000691. doi: 10.1371/journal.pbio.3000691 32218571 PMC7100931

[69] Engzell P, Frey A, Verhagen MD. Learning loss due to school closures during the COVID-19 pandemic. Proceedings of the National Academy of Sciences. 2021; 118(17):e2022376118. doi: 10.1073/pnas.2022376118 33827987 PMC8092566

[70] Eroglu M, Senol C. Emergency Remote Education Experiences of Teachers during the COVID-19 Pandemic: A Phenomenological Research. Shanlax International Journal of Education. 202; 9(3):161–72.

[71] Hebebci MT, Bertiz Y, Alan S. Investigation of views of students and teachers on distance education practices during the Coronavirus (COVID-19) Pandemic. International Journal of Technology in Education and Science. 2020; 4(4):267–82.

[72] Kuhfeld M, Soland J, Tarasawa B, Johnson A, Ruzek E, Liu J. Projecting the potential impact of COVID-19 school closures on academic achievement. Educational Researcher. 2020; 49(8):549–65.

